# Characterization of the serum levels of Meteorin-like in patients with inflammatory bowel disease and its association with inflammatory cytokines

**DOI:** 10.1186/s12944-020-01404-6

**Published:** 2020-10-30

**Authors:** Afsane Gholamrezayi, Maryam Mohamadinarab, Pegah Rahbarinejad, Soudabeh Fallah, Shekufe Rezghi Barez, Leila Setayesh, Nariman Moradi, Reza Fadaei, Elham Chamani, Tahmine Tavakoli

**Affiliations:** 1 Department of Nutrition, School of Public Health–International Campus, Iran University of Medical Sciences, Tehran, Iran; 2 Department of Nutrition, Science and Research Branch, Islamic Azad University, Tehran, Iran; 3 Department of Clinical Biochemistry, Faculty of Medicine, Iran University of Medical Sciences, Tehran, Iran; 4 Department of Clinical Biochemistry, School of Pharmacy and Pharmaceutical Sciences, Isfahan University of Medical Sciences, Isfahan, Iran; 5 Department of Community Nutrition, School of Nutritional Sciences and Dietetics, Tehran University of Medical Sciences (TUMS), Tehran, Iran; 6 Cellular and Molecular Research Center, Research Institute for Health Development, Kurdistan University of Medical Sciences, Sanandaj, Iran; 7 Sleep Disorders Research Center, Kermanshah University of Medical Sciences, Kermanshah, Iran; 8 Cardiovascular Diseases Research Center, Department of Clinical Biochemistry, Birjand University of Medical Sciences, Birjand, Iran; 9 Cardiovascular Diseases Research Center, Department of Internal Medicine, Gastroenterology Section, Faculty of Medicine, Birjand University of Medical Sciences, Birjand, Iran

**Keywords:** Meteorin-like, Inflammatory bowel disease, Ulcerative colitis, Crohn’s disease, Interleukin, Tumor necrosis factor, Adiponectin

## Abstract

**Background:**

Meteorin-like (Metrnl) is an adipokine with insulin sensitizing and anti-inflammatory properties that has been discovered recently. The relation among Metrnl, Inflammatory Bowel Disease (IBD), and obesity has been unexplored yet.

**Methods:**

The present study was conducted on 54 healthy control, 42 Ulcerative Colitis (UC), and 43 Crohn’s disease (CD) patients who were diagnosed by pathological examination. In all participants, serum levels of adiponectin, Metrnl, interleukin (IL)-6, and Tumor necrosis factor (TNF-α) were measured using ELISA kits.

**Results:**

Metrnl concentration was considerably lower in both UC (85.25 ± 36.55 pg/mL) and CD (76.93 ± 27.92 pg/mL) patients in comparison to control (107.52 ± 35.33 pg/mL). In addition, it was seen that both patient groups have a decreased level of adiponectin compared to the controls. Besides that, the level of IL-6 and TNF-α were significantly greater in the patient groups. Moreover, the result showed that the level of Metrnl is inversely correlated with body mass index (BMI) in the controls and the patients. Metrnl levels are also inversely associated with IL-6, and TNF-α in both of the patient groups.

**Conclusions:**

The current study is the first one reporting the decreased levels of Metrnl in serum among patients with IBD, which is inversely related with BMI, TNF-α, and IL-6. These results suggested a possible relation of Metrnl with the pathogenesis of IBD, particularly through inflammatory process, although further studies are warranted to dissect the possible mechanism.

## Background

Inflammatory Bowel Disease (IBD) as one of the causes of mortality in modern societies, characterized by gastrointestinal chronic inflammation [1, 2]. Two most common and pathologic types of IBD are Crohn’s disease and ulcerative colitis (UC). The exact etiology of IBD is not well-understood yet,, therefore no-definitive cure has been identified [3]. In spite of previous research which showed that a possible association of underweight and malnutrition with the occurrence of IBD, recent studies have revealed about 15–40% of patients with IBD are obese and 20–40% are overweight which might contribute to the development of IBD [4]. Recent epidemiological studies have also shown that incidence of IBD is rising in parallel with obesity prevalence [4, 5]. Moreover, the rate of hospitalization and surgery are more frequent among IBD patients with obesity [6]. Scientific studies have suggested that, genetics, gut microbiome, and the immune system may play critical roles in IBD, however, this has not completely understood yet [7]. IBD (UC and CD) also displays the characteristics of chronic inflammation and metabolic syndrome, that induces effective changes in metabolism [8, 9]. Adipocytokines are secreted by white adipose tissue which have effect on the gut microbiome, inflammation, and metabolism pathways [10, 11]. In a mutual way, IBD also can be considered as a risk factor for obesity via changing in the intestinal microbial metabolism [9, 12].

On the other hand, the impaired white adipose tissue (WAT) function such as abnormal adipocytokine secretion has a major and effective role on the inflammatory condition in colon tissue [12]. In this way, it was observed that, leptin concentration was decreased in the serum sample obtained from the IBD patients (with or without overweight). While the levels of resistin, adiponectin, and active ghrelin were remarkably increased [13].

Meteorin-like (Metrnl, known as Subfatin) is a novel adipo-myokine that is mainly expressed in WAT, however, it was reported that Metrnl is also expressed in colon epithelium. The anti-inflammatory function of Metrnl has been revealed recently and also has other roles including enhancing lipid metabolism, decreasing adipose inflammation, and ameliorating obesity-mediated insulin resistance (IR) [14]. Notably, Metrnl expression was higher in mesenteric WAT of the CD patients in comparison to the controls [15]. Metrnl is highly expressed in intestinal cells, white adipose tissue, and skin and also expressed in other tissues including muscle, liver, heart, spleen, and central nervous system (CNS). Moreover, the activated macrophages produced Metrnl, which by this fact, it may be connected to the inflammatory disorders such as IBD [16]. A few studies have been conducted on tissue Metrnl levels in IBD disease. So far, no studies have been performed on investigating the association between serum Metrnl and IBD disease as well as the association among this protein, obesity, and the pro-inflammatory cytokines. Regarding to the fact that IBD is an inflammatory disease and Metrnl anti-inflammatory activities, in this study, the levels of Metrnl in serum were examined in the patients with inflammatory bowel disease and also its association was assessed with the hall-markers of inflammatory cytokines, interleukin (IL)-6, and Tumor necrosis factor (TNF-α).

## Methods

### Study population

This case-control study was conducted on 54 control subjects (42 male and 12 female) and 85 IBD patients including 42 UC (31 male and 11 female) and 43 CD (28 male and 15 female) who were recruited from endoscopy unit of Valiasr Hospital, Birjand, Iran. All the individuals aged between 35 and 60 years old. The patient and control groups were selected by clinical examination, radiologic, endoscopic, and pathologic criteria. Normal people without any inflammatory diseases was considered as control group and based on age, gender and BMI were matched with patient groups. The inclusion criteria for patients were as following; were diagnosed by radiologic, endoscopic examination according to clinical and pathological guideline documents, and presence of other IBD’s manifestations including diarrhea, abdominal pain, rectal bleeding, and malnutrition. In addition, none of them were received medication or anti-inflammatory drugs. All of patients were diagnosed recently. Written consent form was obtained from all participants. The diagnosis of UC/CD were based on established clinical and histopathological criteria. Moreover, the subjects were excluded with any history of cancer, diabetes, autoimmune diseases or active infectious disease.

### Anthropometric data and laboratory measurements

Demographic data and medical history were obtained by a self-questionnaire from all participants. At the beginning of examinations, weight and height were taken from participants who were wearing light clothes, without shoes. Body mass index (BMI) was also calculated by body weight (kg) divided by height squared (m2), and studied targets were categorized into normal weight (BMI < 25) and overweight (BMI ≥ 25). Systolic and diastolic blood pressures of all the participants were measured using a standard sphygmomanometer after 15 min resting in a sitting posture. After 12-h of fast, 5 mL of venous blood was obtained from all the participants and the serum was separated by centrifugation. Subsequently, fasting blood sugar (FBS) and lipid profiles including triglycerides (TG), total cholesterol (TC), High-density lipoprotein-cholesterol (HDL-C), and Low-density lipoprotein-cholesterol (LDL-C) were measured using auto-analyzer and the commercially available kits (Pars Azmoon, Tehran, Iran).

### Serum adipokine and cytokines

Circulating levels of Metrnl were evaluated using an immunoassay kit (R&D Systems, Minneapolis, USA, Cat#DY7867). Moreover, the inter-assay and intra-assay variations were calculated as 6 and 8%, respectively. Adiponectin serum levels were measured using an ELISA kit (Adipogen, Seoul, South Korea, Cat#AG-45A-0001YEK-K101) with inter- and intra-assay variations of 4.4 and 4.6%, respectively. Afterward, the ultrasensitive ELISA kits were used to measure the serum levels of inteleukin-6 (IL-6) (R & D Systems, Minneapolis, USA, Cat# HS600B) and TNF-α (R & D Systems, Minneapolis, USA, Cat# DTA00C). Notably, the minimum detectable ranges of IL-6 and TNF-α were obtained as 0.7 and 1.6 pg/mL. Inter and intra-assay variations of IL-6 were 9 and 7% and inter and also intra-assay variations of TNF-α were 6 and 5%, respectively.

### Statistical analysis

Statistical analysis was performed using SPSS version 18. Categorical data was analyzed using chi-square test and presented in frequency and percentage. Continuous variables were also examined by student t-test and one-way ANOVA, and presented in mean and standard deviation (SD). Pearson correlation test was applied to correlation analysis. Furthermore, multinomial logistic regression was conducted to estimate the risk of diseases status according to serum levels of Metrnl.

## Results

### Anthropometric and biochemical measurement

The anthropometric and biochemical variables of the studied population are presented in Table 1. There is no significant difference in terms of age, sex, and BMI. In addition, there were no significant difference in the frequency of normal weight and overweight between the groups. Although FBS illustrated no significant difference between the controls and patients with UC and CD, insulin and homeostatic model assessment for insulin resistance (HOMA-IR) were dramatically higher in the CD patients compared to the controls. It should be noted that, higher levels of insulin and HOMA-IR in the UC patients compared to the controls did not reach to the significant threshold. Furthermore, lipids profile including TG, TC, HDL-C, and LDL-C and systolic blood pressure (SBP) and diastolic blood pressure (DBP) demonstrated no considerable variation between the patients and controls.

**Table 1:** Anthropometric and biochemical characteristic of studied population

	Control (*n* = 54)	UC (*n* = 42)	CD (*n* = 43)	*P* value
Sex (male/female)	42/12	31/11	28/15	0.373
BMI (kg/m2)	24.12 ± 3.56	23.45 ± 3.64	24.43 ± 4.61	0.502
Normal weight / over weight	29/25	26/16	21/22	0.473
Age (year)	39.02 ± 4.6	38.24 ± 5.32	38.74 ± 5.7	0.763
SBP (mmHg)	131.17 ± 20.79	133.40 ± 24.26	131.65 ± 23.97	0.887
DBP (mmHg)	81.63 ± 13.11	83.10 ± 14.48	83.16 ± 14.26	0.825
FBG (mg/dL)	90.26 ± 9.03	93.65 ± 13.27	94.28 ± 12.05	0.172
Insulin (μU/mL)	4.03 ± 0.31	5.66 ± 0.5	5.93 ± 0.64^b*^	0.014
HOMA-IR	0.89 ± 0.07	1.31 ± 0.14	1.43 ± 0.17^b**^	0.005
TG (mg/dL)	117.41 ± 44.83	128.76 ± 47.64	126.08 ± 38.15	0.409
TC (mg/dL)	157.22 ± 35.41	164.54 ± 47.49	162.25 ± 30.83	0.629
LDL-C (mg/dL)	94.70 ± 26.95	100.64 ± 35.84	104.93 ± 21.74	0.212
HDL-C (mg/dL)	43.53 ± 6.20	42.11 ± 9.07	41.46 ± 7.76	0.394
Adiponectin (μg/mL)	10.29 ± 3.35	7.88 ± 2.83^a**^	7.44 ± 2.44^b**^	< 0.001
TNF-α (pg/mL)	16.08 ± 5.50	29.62 ± 7.40^a**^	30.43 ± 8.25^b**^	< 0.001
IL-6 (pg/mL)	6.38 ± 3.51	8.28 ± 4.11	9.17 ± 4.18^b**^	0.002
Metrnl (pg/mL)	107.52 ± 35.33	76.93 ± 27.92^a**^	85.25 ± 36.55^b**^	< 0.001

Data are presented as Mean ± SD

One way ANOVA and Tukey’s multiple comparisons test were used for comparison of quantitative variables

*Abbreviations*: *UC* ulcerative colitis, *CD* Crohn’s disease, *SBP* systolic blood pressure, *DBP* diastolic blood pressure, *FBG* fasting blood glucose, *HOMA-IR* homeostasis model assessment of insulin resistance, *LDL-C* low-density lipoprotein cholesterol, *HDL-C* high-density lipoprotein cholesterol, *TC* total cholesterol, *TG* triglyceride

^a^ Comparison between control and UC

^b^ Comparison between control and CD

*P < 0.05

**P < 0.01

### Serum levels of adipokines and cytokines

**Fig. 1:**
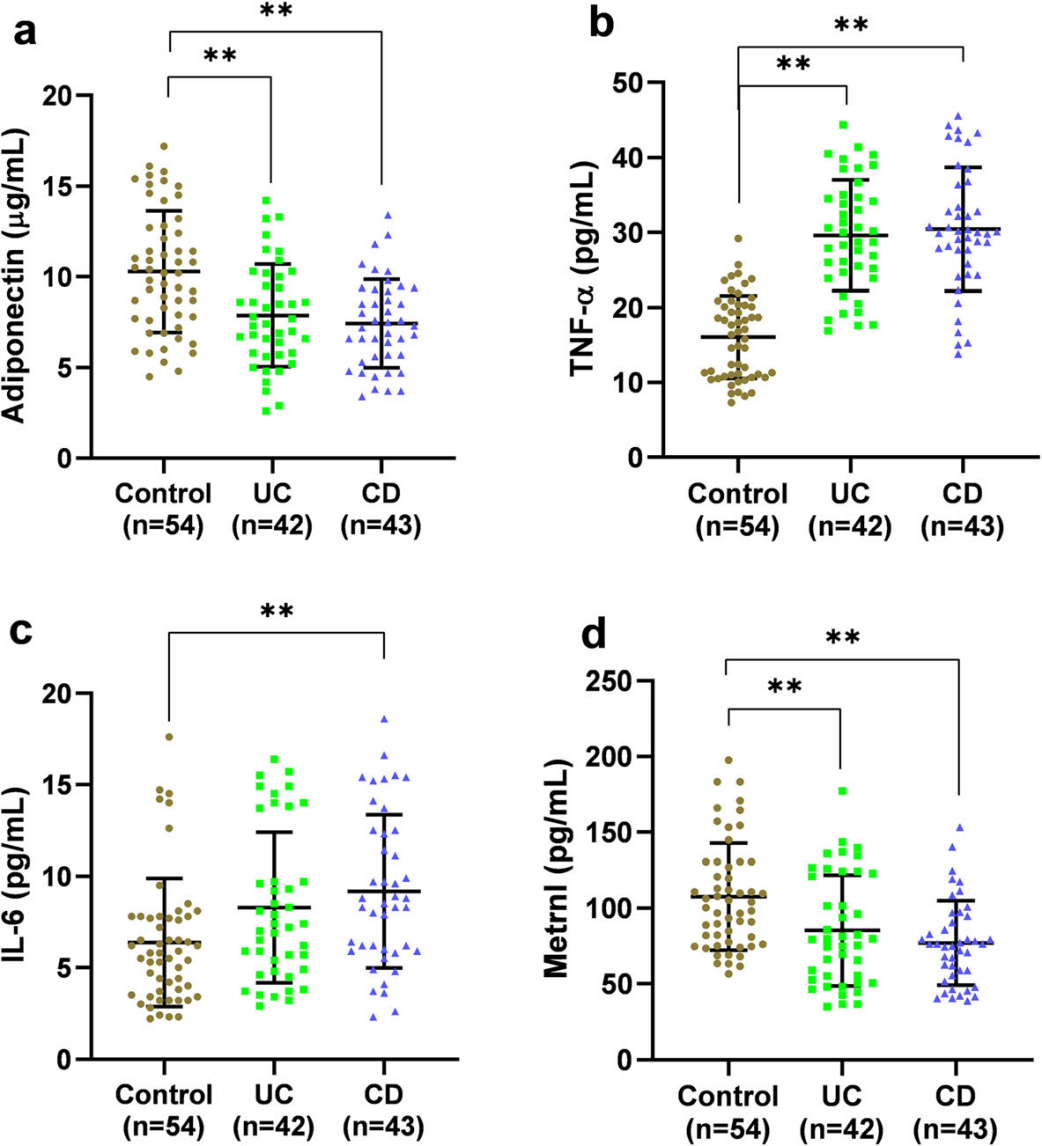
Serum levels of adipokines and cytokines. **a** Serum levels of adiponectin decreased significantly in both UC and CD patients compared to controls. **b** Serum levels of TNF-alpha were found to be lower in both patient groups compared to controls. **c** IL-6 serum concentration indicated a considerable increase in CD group compared to controls. **d** Metrnl serum levels demonstrated a significant decline in UC and CD patients compared to controls. CD, Crohn’s disease; IL-6, Interleukin 6; TNF-α, Tumor Necrosis Factor Alpha; UC, ulcerative colitis

The ELISA results (Fig. 1) showed that, adiponectin concentration was considerably lower in the patients with UC and CD compared to the controls. Furthermore, an elevated level of TNF-α in the patients in comparison to the controls was observed. Moreover, patients with CD showed higher IL-6 levels compared to the controls, while this figure for the serum levels of IL-6 did not reach to the significant threshold in the patients with UC. Furthermore, a decreased concentration of Metrnl serum was observed in the patients with UC and CD compared to the controls.

Regarding the crucial role of adipose tissue on Metrnl levels, analysis was performed according to the BMI cutoff (Overweight, BMI ≥ 25 and normal weight, BMI < 25). In this regard, Metrnl serum levels were found to be lower in all the overweight subgroups (Table 2).

**Table 2:** Serum levels of Metrnl according to BMI cutoff

Group	Normal weight	Overweight	*P*
All participants	102.27 ± 36.23	78.13 ± 31.04	< 0.001
Control	117.84 ± 37.53	95.56 ± 28.90	0.019
UC	96.92 ± 35.63	66.29 ± 30.25	0.007
CD	87.40 ± 27.24	66.93 ± 25.24	0.014

Data are presented as Mean ± SD

ANOVA test was used to assess Metrnl levels, among three groups based on BMI cutoff (Overweight, BMI ≥ 25 and normal weight, BMI < 25)

*Abbreviations*: *UC* ulcerative colitis, *CD* Crohn’s disease

### Association of serum Metrnl with the risk of diseases status

Multinomial logistic regression was performed to assess the risk of diseases status according to serum levels of Metrnl. The results demonstrated a significant association between the decreased levels of Metrnl with the risk of UC and CD diseases. Furthermore, these associations have been adjusted for confounding factors including age, sex and BMI and the relationships remained as significant for both UC and CD diseases (Table 3).

**Table 3:** Odd ratio of diseases status according to 10 unit change in the serum levels of Metrnl

Model	Group	Odd Ratio (95% CI)	β (standard error)	*P* value	Correct prediction (%)
Crude	UC	0.833 (0.735–0.944)	− 0.183(0.064)	0.004	57.1
	CD	0.760 (0.661–0.874)	− 0.275(0.071)	< 0.001	69.8
Model 1	UC	0.794 (0.692–0.912)	− 0.230(0.070)	0.001	57.1
	CD	0.738 (0.636–0.858)	− 0.303(0.076)	< 0.001	58.1

*Abbreviations*: *UC* ulcerative colitis, *CD* Crohn’s disease, *CI* Confidence interval

Model 1. Adjusted for age, sex and BMI

### Correlation analysis

**Fig. 2:**
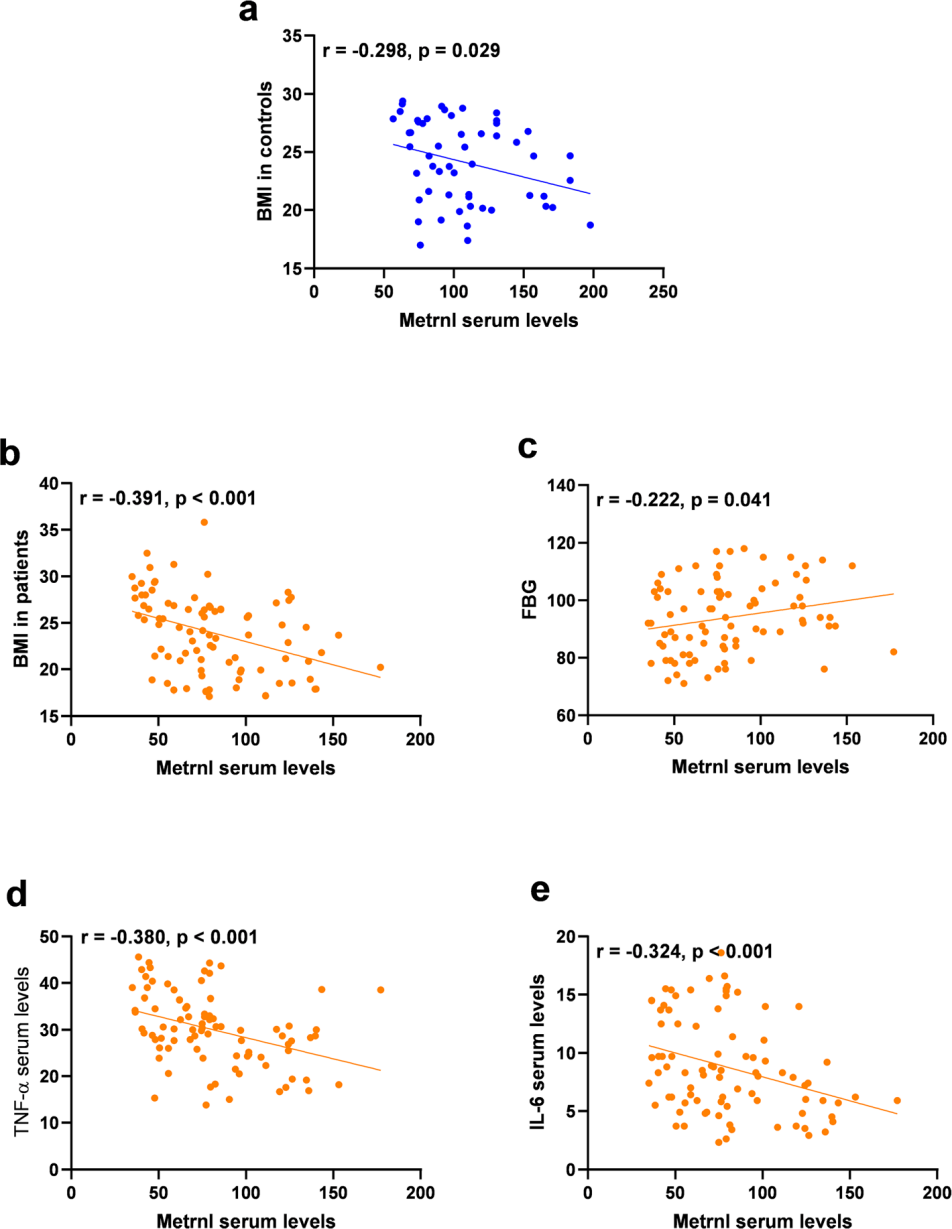
The correlation of Metrnl with **a**) BMI in controls, **b**) BMI in patients, **c**) FBG in patients, **d**) TNF-α in patients and **e**) IL-6 in patients. BMI, body mass index; TNF-α, Tumor Necrosis Factor Alpha; IL-6, Interleukin 6

Correlation analyses were performed in 2 subgroups, as controls and patients and the results are presented in Table 4. In the control group, Metrnl was found to be inversely correlated with BMI (Fig. 2). Metrnl also had an inverse correlation with BMI, IL-6, and TNF-α, and a positive correlation with FBG in the patient groups (Fig. 2).

**Table 4:** Correlation analysis of serum Metrnl levels with anthropometric and biochemical variables

	Metrnl	
	Control	IBD
BMI (kg/m2)	−0.298^*^	− 0.391**
Age (year)	0.174	−0.146
SBP (mmHg)	−0.026	0.032
DBP (mmHg)	−0.110	−0.061
FBG (mg/dl)	0.023	0.222*
Insulin (uU/ml)	−0.184	0.085
HOMA-IR	−0.175	0.127
TG (mg/dl)	0.202	−0.004
TC (mg/dl)	0.082	−0.016
LDL-C (mg/dl)	0.026	−0.061
HDL-C (mg/dl)	0.085	−0.029
Adiponectin (ug/ml)	−0.012	−0.076
TNF-α (pg/mL)	0.137	−0.380**
IL-6 (pg/ml)	−0.027	−0.324**

*Abbreviations*: *UC* ulcerative colitis, *CD* Crohn’s disease, *SBP* systolic blood pressure, *DBP* diastolic blood pressure, *FBG* fasting blood glucose, *HOMA-IR* homeostasis model assessment of insulin resistance, *LDL-C* low-density lipoprotein cholesterol, *HDL-C* high-density lipoprotein cholesterol, *TC* total cholesterol, *TG* triglyceride

*P < 0.05

**P < 0.01

## Discussion

Adipokines, have regulatory roles on expression and secretion of various cytokines, by this fact they have significant effects on the immune system. Therefore, they can play a crucial role in inflammatory diseases like IBD, which also have a metabolic background [17]. Several studies have shown that, adipokines such as leptin, resistin, visfatin, retinol-binding protein-4, adiponectin, glucose, and insulin are deregulated in the IBD patients [13]. Metrnl is a novel adipokine, which has a major role in improvement of inflammation and insulin resistance improvement [18]. Accordingly, this adipokine has been investigated in several metabolic and inflammatory diseases. Lee et al. showed that, patients with diabetes mellitus type 2 (T2DM) had lower level of Metrnl in their serum [19]. Similarly, Dadmanesh et al. found that Metrnl concentration in the serum of patients with coronary artery disease and T2DM were lower [20]. While, Chung et al. reported that levels of Metrnl were significantly higher in T2D patients [21]. Results of *Wanng* et al, also represented increase in serum level of Metrnl in T2M patients in comparison to control. They proposed increase in serum Metrnl could strengthen the risk of T2DM independent of insulin resistance [22]. This contradiction might be a results from the difference in study population, diabetes duration and medication. Most of the previous studies have focused on the tissues level of Metrnl in inflammatory disorders, thus there is no data on the serum levels of Metrnl in these complications. Bridgewood et al. investigated the level of Metrnl in synovial tissue in the patients with Rheumatoid Arthritis, Psoriatic Arthritis, and Osteoarthritis. As a result, they found the elevated level of Metrnl in Psoriatic Arthritis [23]. To the authors knowledge, so far, current study is the novel one that reporting the serum levels of Metrnl in the IBD patients. In addition, the results show the lower serum levels of Metrnl in the IBD patients compared to the controls. However, Metrnl was not different between patients groups. Li et al. demonstrated that, Metrnl is highly expressed in the gastrointestinal tract of normal donors as well as mice. On the other hand, they produced intestinal epithelial cell-specific knockout mice, in spite of reduction in Metrnl expression in the gastrointestinal tract, it’s level is not decreased in serum [16]. A recent study performed by Zuo et al. reported that Metrnl expression is higher in mesenteric adipose tissue (MAT) of the CD patients compared to the controls. They also showed that, systemic treatment of Metrnl can improve the adipocyte function, and reduce the macrophage infiltration and inflammation by acting on the peroxisome proliferator-activated receptors (PPARγ) pathway in mice. Therefore, they suggested that, upregulation of Metrnl in the MAT of the patients may be a compensatory response [14]. Regarding the inconsistent results, it seems likely that, Metrnl expression can have an organ dependent pattern; however, further longitudinal research is needed to support this hypothesis.

Furthermore, present study indicated an inverse relationship of Metrnl with the inflammatory cytokines in the IBD patients. It was also observed that, Metrnl may have regulatory action in inflammation pathways. Zuo et al. administered the Metrnl in IL-10−/− mice and then observed a significant decrease in the score of inflammation and pro-inflammatory factors such as TNF-α, interferon (IFN)-γ, and IL-6 [12]. Additionally, Zhi-yong LI et al. reported that, Metrnl plays a regulatory role in the expression of antimicrobial peptides such as islet-derived 3 gamma (Reg3g), lactotransferrin, and amyloid A-3 (SAA3) [23]. Since TNF-α and IL-6 are considered as the markers of inflammation, the results suggest a relationship between Metrnl and IBD pathogenesis. In addition, adiponectin decreased in the patient’s groups, however, there were no relation between Metrnl and adiponectin that suggested a different regulation of these two adipokines.

When the population were stratified based on obesity, the serum level of Metrnl was significantly lower in obese subjects than in non-obese ones. Consistently, AlKhairi et al. reported that, Metrnl is significantly higher in the T2DM obese patients, in a way that this elevation can be explained as a compensatory response [24]. However, Zhi-Yong Li et al. showed no correlation between serum Metrnl levels and BMI [25]. As the adipose tissue is the main source of Metrnl secretion, it is expected that, BMI can affect the levels of this adipokine, and adipose tissue inflammation and dysfunction may be considered as the causes for the decrease in Metrnl levels.

### Study strength and limitations

The present study for the first time measured Metrnl serum levels in patients UC and CD disease and its relation with inflammatory cytokines. The studied population were matched in terms of age, sex and BMI with control group that eliminated the impact of these confounding factor on the results. On the other hand, the present study has no data on body fat distribution that could be more clinically significant than BMI. Furthermore, the cross-sectional design of the study limited us in concluding a cause and effect relationship, so further studies are waranted to dissect the possible mechanism for the reported relationship.

## Conclusion

In conclusion, the current study for the first time showed that a decreased level of Metrnl in the serum of IBD patients. Moreover, it was found that, serum level of Metrnl has a negative correlation with levels of TNF-α, IL-6 and BMI in the serum of patients with IBD. Altogether, the present study found that Metrnl, inflammation, and obesity are related. This finding may suggest a possible association of Metrnl with the pathogenesis of IBD which can be considered for ameliorating the inflammatory milieu in these patients.

## Data Availability

Additional data are available from the corresponding authors for reasonable requesting.

## Abbreviations

Metrnl: Meteorin-like
IBD: Inflammatory bowel disease
CD: Crohn’s disease
IL-6: Interleukin 6
TNF-α: Tumor necrosis factor α
WAT: White adipose tissue
TG: Triglyceride
Chol: Cholesterol
FBS: Fasting blood sugars
SD: Standard deviations
T2DM: type 2 diabetes mellitus
HOMA-IR: Homeostatic model assessment- insulin resistance
SBP: Systolic blood pressure
DPB: Diastolic blood pressure
